# Gender-specific effects of meat consumption on type 2 diabetes: a narrative review of prospective cohort studies

**DOI:** 10.3389/fnut.2025.1665566

**Published:** 2025-11-21

**Authors:** Mauro Lombardo

**Affiliations:** Department for the Promotion of Human Science and Quality of Life, San Raffaele Open University, Rome, Italy

**Keywords:** type 2 diabetes, red meat, processed meat, gender differences, prospective cohort studies, dietary risk factors, meat consumption

## Abstract

**Purpose of the review:**

This narrative review examines the association between meat consumption and the risk of type 2 diabetes mellitus (T2DM), with a focus on gender-specific effects.

**Results:**

Prospective cohort studies indicate that processed and red meat consumption is associated with an increased risk of T2DM, with stronger associations generally observed in men. In contrast, white meat shows weaker and inconsistent associations, with modest increases in risk reported more often in women.

**Summary:**

The evidence supports the relevance of gender in dietary recommendations for the prevention of T2DM. Biological and behavioral differences, such as hormonal profiles, iron metabolism, fat distribution and culinary habits, may underlie sex-specific susceptibilities. Personalized dietary strategies should take these differences into account to improve prevention.

## 1 Introduction

The relationship between meat consumption and metabolic health has been widely debated, with numerous epidemiological studies suggesting an association between high intake of red and processed meat and an increased risk of type 2 diabetes (T2DM) (1–4). T2DM is a chronic metabolic disorder characterized by insulin resistance and hyperglycaemia, leading to severe complications such as neuropathy, nephropathy and cardiovascular disease (5, 6). While dietary patterns are known to influence T2DM risk, the specific role of meat consumption in its pathogenesis remains complex and not fully understood. Several mechanisms have been proposed to explain the link between meat consumption and T2DM risk (7). Processed meats contain high levels of sodium, nitrates and advanced glycation end-products (AGEs), which have been implicated in oxidative stress, chronic inflammation and pancreatic β-cell dysfunction, key factors in the development of insulin resistance (8). It should be noted that, while observational studies suggest a positive association between high intake of red and processed meat and increased risk of T2DM, randomized controlled trial evidence is less consistent. A recent meta-analysis of RCTs found no significant effect of total red meat intake on glycaemic control or inflammatory biomarkers (9).

An emerging area of research emphasizes the importance of gender differences in metabolic response to diet. Men and women have distinct physiological, hormonal and metabolic traits that may modulate their susceptibility to T2DM (10, 11). Men are more prone to visceral fat accumulation and insulin resistance, whereas women undergo metabolic changes influenced by hormonal fluctuations, particularly during menopause, which may alter their T2DM risk profile (12). Despite these known differences, most existing reviews and meta-analyses have not examined whether and how men and women differ in their metabolic response to different types of meat. This creates a critical gap in the literature, which our review aims to fill by providing a focused synthesis of prospective cohort studies reporting gender-specific associations.

This narrative review aims to provide an up-to-date summary of the evidence on the association between meat consumption and T2DM, with a focus on gender differences. By examining available data from epidemiological studies, this review seeks to clarify whether men and women are affected differently by meat consumption in relation to T2DM risk, thus contributing to a more nuanced understanding of dietary recommendations for metabolic health.

## 2 Methods

### 2.1 Literature search and eligibility criteria

This narrative review was conducted following the SANRA (Scale for the Assessment of Narrative Review Articles) guidelines (13), to ensure transparency, methodological rigor, and comprehensive reporting. Literature searches were performed in PubMed and Scopus, combining terms related to meat intake (“red meat”, “processed meat”, “unprocessed meat”, “white meat”, “poultry”) and metabolic outcomes (“type 2 diabetes”, “insulin resistance”, “glucose intolerance”) with Boolean operators adapted to each database. No date restrictions were applied. The last search was performed in August 2025. Eligible studies were prospective cohort investigations that assessed the association between meat consumption and T2DM, provided hazard ratios with corresponding confidence intervals, and reported results either stratified by sex or included an interaction term by sex. Cross-sectional studies, reviews, and articles without incidence or hazard ratio data were excluded. Only studies reporting sex-specific risk estimates were included, while those presenting pooled results without sex disaggregation were not considered, regardless of sample size or relevance. Supplementary material were reviewed when available to confirm effect estimates and subgroup definitions. Exposures were reported heterogeneously (quintiles, servings/day, cooking methods). No harmonization was attempted, in order to preserve methodological detail and avoid speculative standardization. Results are therefore presented descriptively, and direct comparisons require caution.

### 2.2 Handling of overlapping cohorts

To avoid the risk of data duplication, special attention was given to studies based on the Nurses' Health Study (NHS, initiated in 1976), the Nurses' Health Study II (NHS II, initiated in 1989), and the Health Professionals Follow-up Study (HPFS, initiated in 1986), which are widely used in epidemiological research. Only unique risk estimates for each cohort, gender and analytical period were considered, and in cases where multiple publications reported overlapping results, the most recent or comprehensive analysis was selected. The results of these cohorts were included only if data for male and female populations were reported in the same publication, thus allowing a direct comparison by sex under consistent methodological conditions. Single-sex cohorts published in isolation, without a counterpart of the opposite sex in the same study, were not considered in order to avoid indirect or methodologically inconsistent comparisons.

### 2.3 Exposure definitions

Exposures were classified according to conventional epidemiological definitions. Total meat (TM) included the sum of red (RM), processed (PM), and white meat (WM). RM (red meat) refers specifically to unprocessed beef, pork, lamb, or veal, while PM (processed meat) includes products preserved by smoking, curing, salting, or the addition of chemical preservatives (such as sausages, salami, ham, bacon, and hot dogs). WM refers to unprocessed poultry, particularly chicken and turkey. When original studies reported “total red meat” (TRM), including both processed and unprocessed forms, this was explicitly indicated in the tables and figures.

### 2.4 Data extraction and synthesis

**Figure 1:**
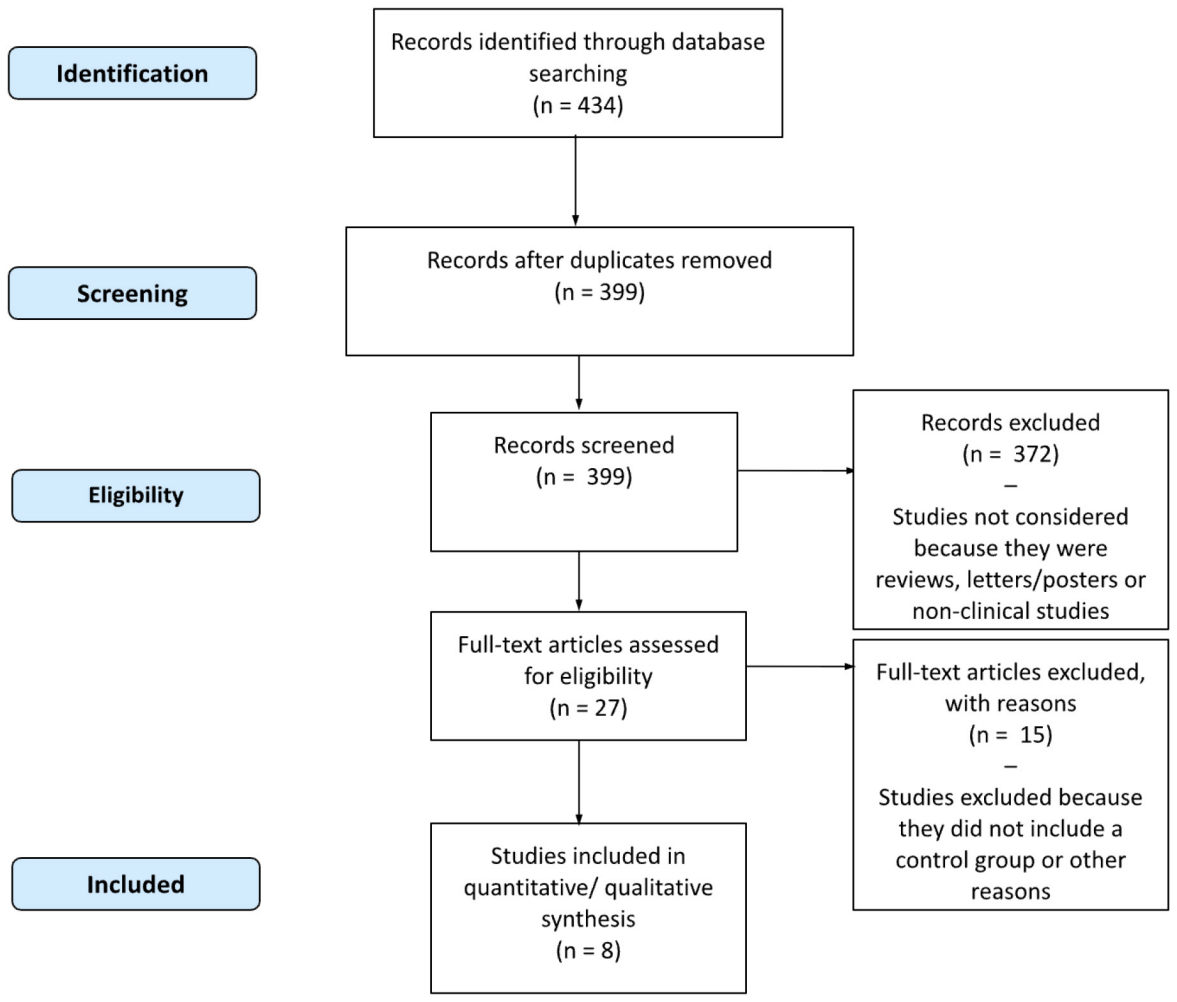
Flowchart of study selection for inclusion in the narrative review.

For each study that met the inclusion criteria, details regarding the author, year, country, population characteristics, meat exposure, results, follow-up duration, sex-specific risk ratios with confidence intervals, and overall interpretation of the association were collected and verified. The study selection process is illustrated in Figure 1, and methodological details are summarized in Supplementary Table S1, including exposure contrasts, covariates in the fully adjusted models, and effect measure type (odds ratio or hazard ratio). Given the narrative nature of this review, hazard ratios derived from heterogeneous exposure contrasts (e.g., quintiles, servings/day, 50–100 g/day, or cooking frequency) were reported as originally presented, without harmonization or dose re-expression. This decision preserved methodological integrity and avoided speculative standardization, but necessarily reduces comparability across studies. Multiple publications analyzing the NHS I, NHS II, and HPFS cohorts were included because they addressed distinct methodological questions: Pan et al. examined associations fully adjusted for BMI; Gu et al. excluded BMI to explore mediation by adiposity; and Liu et al. focused on cooking methods and doneness levels, adjusting for total meat intake and BMI. These were treated as complementary perspectives rather than independent confirmations. Although only studies reporting sex-specific estimates were included, formal interaction tests between sex and meat intake were rarely performed. Reported HRs and 95% CIs were therefore considered as presented; in some cases, overlap of CIs between sexes was noted as a cautious indicator. However, no pooled meta-regression or formal interaction analysis was attempted, in line with the qualitative objectives of this review. Consequently, the results should be interpreted as exploratory, anchored to study-specific exposure definitions and adjustment sets, and not as confirmatory evidence of sex-based effect modification.

Several otherwise relevant prospective cohort studies were excluded because they did not report hazard ratios stratified by sex or did not include an interaction term. This choice was deliberate to ensure comparability of gender-specific estimates across studies.

## 3 Results

Given the heterogeneous exposure metrics across cohorts, between-study contrasts are interpreted qualitatively. To minimize over-interpretation, overlap of 95% confidence intervals between male and female estimates is noted when relevant, acknowledging that this does not provide formal evidence of interaction. Table 1 describes the cohort characteristics of the included studies. The majority were conducted in the USA, with the exception of Ericson et al. (Sweden), Kurotani (Japan), and the EPIC-InterAct Consortium (eight European countries). Most studies included both men and women, although the proportion of female participants was notably higher in Pan et al., Liu et al., and Gu et al., which are based on the NHS and NHS II cohorts. All studies evaluated RM and PM; several also assessed TM (Vang, EPIC-InterAct, Kurotani), WM (EPIC-InterAct, Ericson, Liu), or RM/PM separately (Gu). Follow-up durations ranged from 5 to 28 years.

**Table 1:** Characteristics of the prospective studies included in the review, by exposure type and gender-specific population.

**Authors**	**Year**	**Country**	**Population**		**Exposure**	**Duration (yrs)**	**Ref**.
			**Males**	**Females**			
Vang et al.	2008	USA	5,208	3,193	TM	17	14
Steinbreche et al.	2011	USA	29,759	35,244	RM, PM	14	15
EPIC-InterAct – Consortium	2013	8 European countries	137,000	204,000	TM, RM, PM, WM	11.7	16
Ericson et al.	2013	Sweden	10,550	16,590	RM, PM, WM	12	17
Kurotani	2013	Japan	27,425	36,424	TM, RM	5	18
Pan et al.	2011	USA	37,083	166,074	RM, PM	20–28	19
Liu et al.	2018	USA	24,679	113,561	RM, WM (high-temp cooking)	16	20
Gu et al.	2023	USA	41,172	175,523	PM; RM	up to 28	21

This table summarizes the main characteristics of the prospective cohort studies included in the review that investigated the association between different types of meat consumption and risk of type 2 diabetes (T2D), with data reported separately for males and females where available. Contrast units: Q5 vs. Q1 unless otherwise specified; when exposures were reported as servings/day or g/day, the exact unit is indicated in the text and/or in figure labels. Models are fully adjusted as in the original publications. Statistical significance was defined as two-sided *p* < 0.05. Abbreviations: TM, total meat; RM, unprocessed red meat; PM, processed meat; WM, unprocessed poultry; TRM, total red meat; HR, hazard ratio; OR, odds ratio; CI, confidence interval. The symbol ^*^ indicates that the association refers to meat cooked at high temperatures, not to quantity consumed.

Table 2 summarizes prospective studies investigating the association between meat consumption and T2DM, with results stratified by sex when available. A positive association was more frequently observed among men for RM and PM (3 studies each), while WM was more often associated with increased risk in women (3 studies). For TM, findings were mixed, with a slightly stronger pattern in men (2 studies vs. 1 in women). In several cohorts, associations were similar across sexes or not statistically significant. These results suggest potential gender-specific differences, particularly for RM and PM in men and WM in women.

**Table 2:** Gender-specific associations between meat consumption and type 2 diabetes risk in prospective cohort studies.

**Authors**	**TM**		**RM**		**PM**		**WM**		**ref**.
	**M**	**F**	**M**	**F**	**M**	**F**	**M**	**F**	
Vang et al.	↑	↑							14
Steinbrecher et al.			↑	↑	↑	↑	=	=	15
EPIC-InterAct – Consortium	↑	↑	↑	=	↑	=	=	↑	16
Ericson et al.			=	=	↑	↑	=	↑	17
Kurotani	↑	=	↑	=	=	=	=	=	18
Pan et al.	↑	↑	↑	↑	↑	↑			19
Liu et al.^*^	=	↑	↑	↑			=	↑	20
Gu et al.			↑	↑	↑	↑	↑	↑	21

The table includes prospective studies that analyzed the association between different types of meat consumption and T2DM risk, reporting hazard ratios (HR) separately for men and women when available. All studies used HR, except Vang et al. (39) and Kurotani et al. (38), which reported odds ratios (OR). Contrast units: Q5 vs. Q1 unless otherwise specified; where exposures were reported as servings/day or g/day, the exact unit is indicated in the text/figure label. Models are fully adjusted as in the original publications, with covariates as specified by the study authors. Statistical significance was defined as two-sided *p* < 0.05. The abbreviations used are: TM, total meat; RM, red meat; PM, processed meat; WM, white meat; TRM, total red meat; HR, hazard ratio; OR, odds ratio; CI, confidence interval. The symbol ^*^ indicates that the risk is related to the cooking method and not to the quantity consumed. The arrows represent the direction of the association: ↑ indicates a significant increase in risk (HR or OR with 95% CI fully >1), ↓ a significant decrease, = no association, and ≈ ↑ a non-significant positive trend (confidence interval including 1.00). All values are from fully adjusted multivariate models, as reported by the authors.

**Figure 2:**
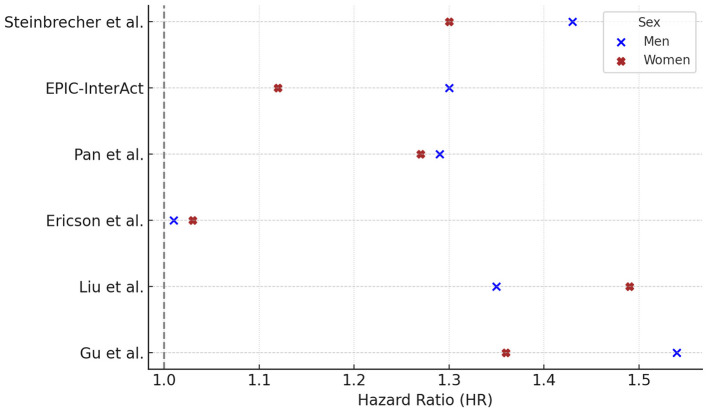
Red meat consumption and type 2 diabetes risk: gender-specific hazard ratios. Hazard ratios (HR) and 95% confidence intervals (CI) for the association between red meat consumption and risk of type 2 diabetes in prospective cohort studies, stratified by sex. Reported HRs (95% CI) for males were: Steinbrecher 1.43 (1.29–1.59), EPIC 1.30 (1.09–1.56), Pan 1.29 (1.11–1.50), Ericson 1.01 (0.82–1.25), Liu 1.35 (1.10–1.66), Gu 1.54 (1.35–1.76); and for females: Steinbrecher 1.30 (1.17–1.45), EPIC 1.12 (0.96–1.30), Pan 1.23 (1.14–1.33), Ericson 1.03 (0.82–1.25), Liu 1.40 (1.23–1.60), Gu 1.36 (1.26–1.47). For Pan et al. (40), HRs reflect comparisons of the highest vs. lowest quintile of intake (Q5 vs. Q1), fully adjusted for BMI and lifestyle factors. For Liu et al. (41), HRs refer to >15 vs. <4 times/month of open-flame/high-temperature cooking, adjusted for total meat intake, BMI, and other covariates. For Gu et al. (42), HRs derive from Model 3, which excluded BMI to explore mediation by adiposity, comparing Q5 vs. Q1 of meat intake. Statistically significant associations were observed in both sexes in Pan et al. (*p* < 0.001) and Liu et al. (*p* = 0.002 in men; p < 0.001 in women). *P*-values were not available for the other studies. Contrast units: Q5 vs. Q1 unless otherwise specified; where exposures were reported as servings/day or g/day, the exact unit is indicated in the text/figure label. Models are fully adjusted as in the original publications (covariates as specified by the authors). Statistical significance was defined as two-sided *p* < 0.05. HRs are presented exactly as reported in the primary studies.

Six prospective cohort studies reported gender-specific HRs for the association between RM consumption and T2DM. Among these, four studies (Steinbrecher, EPIC, Pan, Gu) found higher HRs in men, while two (Ericson, Liu) reported higher HRs in women (Figure 2).

**Figure 3:**
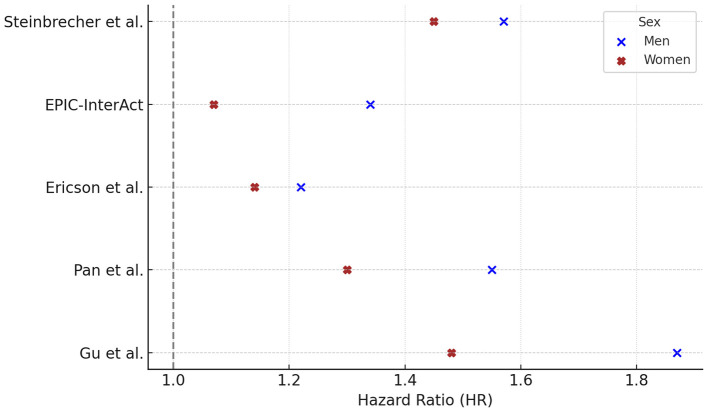
Processed meat consumption and type 2 diabetes risk: gender-specific hazard ratios. Hazard ratios (HR) and 95% confidence intervals (CI) for the association between processed meat consumption and risk of type 2 diabetes in prospective cohort studies, stratified by sex. HRs (95% CI) for males were: Steinbrecher 1.57 (1.42–1.75), EPIC 1.34 (1.14–1.57), Ericson 1.22 (0.99–1.51), Pan 1.55 (1.33–1.79), Gu 1.87 (1.65–2.11); and for females: Steinbrecher 1.45 (1.30–1.62), EPIC 1.07 (0.92–1.25), Ericson 1.14 (0.91–1.43), Pan 1.30 (1.20–1.40), Gu 1.48 (1.38–1.59). For Pan et al. (40), HRs reflect Q5 vs. Q1 contrasts fully adjusted for BMI and lifestyle factors. For Gu et al. (42), HRs are based on Q5 vs. Q1 from Model 3, which excluded BMI to examine potential mediation by adiposity. Statistically significant results were reported in males (Pan *p* < 0.001; Ericson *p* = 0.02) and in females (Pan *p* < 0.001; Ericson *p* = 0.08). *P*-values were not available for Steinbrecher, EPIC, and Gu. Notes. Contrast units: Q5 vs. Q1 unless otherwise specified; when exposures were reported as servings/day or g/day, the exact unit is indicated in the text/figure label. Models are fully adjusted as in the original publications, with covariates as specified by the study authors. Statistical significance was defined as two-sided *p* < 0.05. HRs are presented exactly as reported in the primary studies.

Five prospective studies reported HRs for PM consumption and T2DM, stratified by sex. In all five studies the HR was higher in men than in women, with values ranging from 1.22 to 1.87 in men and from 1.07 to 1.48 in women (Figure 3).

**Figure 4:**
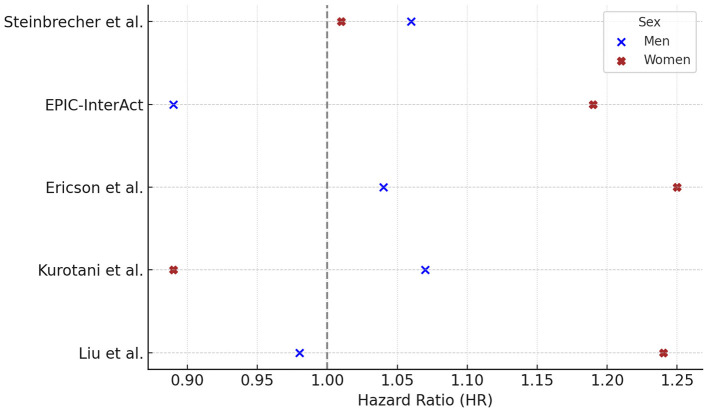
White meat consumption and type 2 diabetes risk: gender-specific hazard ratios. Hazard ratios (HR) and 95% confidence intervals (CI) for the association between white meat consumption and risk of type 2 diabetes in prospective cohort studies, stratified by sex. HRs (95% CI) for males were: Steinbrecher 1.06 (0.96–1.18), EPIC 0.89 (0.76–1.04), Ericson 1.04 (0.85–1.26), Kurotani 1.07 (0.85–1.34), Liu 0.98 (0.66–1.06); and for females: Steinbrecher 1.01 (0.90–1.14), EPIC 1.19 (1.02–1.38), Ericson 1.25 (1.03–1.52), Kurotani 0.89 (0.68–1.15), Liu 1.24 (1.08–1.41). For Liu et al. (41), HRs reflect >15 vs. <4 times/month of open-flame/high-temperature cooking frequency, adjusted for total meat intake, BMI, and other covariates. Statistically significant *p*-values were reported for Ericson (*p* = 0.02) and Liu (*p* = 0.001) in females; *p* = 0.79 for males in Ericson. All other *p*-values were not available. Contrast units: Q5 vs. Q1 unless otherwise specified; when exposures were reported as servings/day, g/day, or cooking frequency, the exact unit is indicated in the text/figure label. Models are fully adjusted as in the original publications, with covariates as specified by the study authors. Statistical significance was defined as two-sided *p* < 0.05. HRs are presented exactly as reported in the primary studies.

Among the five studies reporting gender-specific hazard ratios (HRs) for white meat consumption and T2DM risk, three studies found HRs > 1.0 in both sexes (Steinbrecher, Ericson, Kurotani). In Liu et al., the HR exceeded 1.0 only in women (HR = 1.24), while in EPIC only women showed an HR > 1.0 (HR = 1.19). No study reported an HR > 1.0 for men alone (Figure 4).

## 4 Discussion

### 4.1 Overview of scientific evidence on meat consumption and type 2 diabetes

The link between meat consumption and type 2 diabetes remains a central theme in nutritional epidemiology. Data collected from large cohorts in Europe, the United States and Asia indicate that high consumption of red and processed meat is generally associated with an increased risk of type 2 diabetes, while the results for white meat are weaker and less consistent (14–16). A recent federated meta-analysis, which brought together nearly two million individuals from 31 cohort studies, confirmed a modest but significant increase in diabetes risk across all meat categories (17). Within this general picture, some prospective cohort studies suggest possible sex-specific differences, notably stronger associations for processed and red meat in men and more variable patterns for white meat in women. However, aggregate analyses based on individual participant data found no statistical evidence of interaction between the sexes (17). This underscores the importance of interpreting observed differences between the sexes as signals rather than definitive conclusions, while recognizing potential biological and behavioral explanations, such as differences in hormonal profiles, fat distribution, iron metabolism, and dietary habits. Regional dietary cultures also influence these associations. In Asian populations, where red meat consumption is relatively low and diets include more fish and soy products, the reported associations with diabetes risk are often weaker and sometimes vary by sex (18). In contrast, cohorts from Europe and North America, characterized by higher consumption of red and processed meat and lower consumption of protective plant foods, tend to show stronger and more consistent associations, especially in men. These cultural and dietary contexts provide an important background for understanding where and why gender-specific risk patterns may emerge. Overall, current evidence suggests that meat consumption contributes to the risk of type 2 diabetes in different populations, with possible but as yet unconfirmed differences between men and women. Exploring these patterns in relation to both biological mechanisms and regional dietary environments remains a key direction for future research.

### 4.2 Biological and behavioral drivers of sex differences in meat-related diabetes risk

Several biological mechanisms may explain the stronger associations observed in men. Estrogen exerts insulin-sensitizing and anti-inflammatory effects, providing some metabolic protection in premenopausal women (19). In contrast, men, with lower estrogen levels, may be more sensitive to the pro-inflammatory effects of nitrates, nitrites, haem iron and AGEs present in processed meats (20–22). Men also tend to have more visceral adipose tissue and greater iron stores, both of which are linked to increased insulin resistance and oxidative stress (23, 24). Men often consume larger portions of RM and PM and fewer protective foods such as vegetables and legumes. This dietary pattern increases exposure to harmful compounds and reduces antioxidant intake (25). Social and cultural gender roles may reinforce these patterns, as demonstrated by the HELIUS (18) and Swiss cohort (19) studies, potentially increasing metabolic risk. Cultural norms also shape gendered eating behaviors, with men in Western settings more likely to consume larger portions of red and processed meat, whereas women often report higher poultry intake. These sociocultural factors may reinforce biological susceptibilities, contributing to the heterogeneous risk patterns observed across regions.

### 4.3 Metabolic pathways and sex-related effects

RM contributes to the intake of heme iron, which can promote oxidative stress and beta cell dysfunction, particularly in men (26). It also affects circulating metabolomic profiles, increasing BCAAs and reducing glycine, both of which may be linked to insulin resistance (27). In addition, RM is a dietary source of L-carnitine, which is converted to TMAO, a compound that may be associated with inflammation and metabolic dysfunction (28, 29). Heterocyclic amines (HCAs), such as PhIP and harman, are mutagenic compounds formed during high-temperature cooking of red and processed meats, particularly grilling and frying (30). These compounds can impair insulin signaling by increasing hepatic glucose production, interfering with insulin-induced AKT phosphorylation, and promoting gluconeogenic gene expression (31, 32). Men may be more susceptible to these effects due to lower estrogen levels and genetic differences in HCA metabolism, especially involving N-acetyltransferase 2 (NAT2) polymorphisms, which modulate the detoxification capacity and influence metabolic response to HCAs (33). A Finnish cohort study showed that partial replacement of red or processed meat with plant-based foods moderately reduced the risk of T2DM (HR = 0.97, 95% CI: 0.95–1.00) (34). White meat shows less consistent associations with T2DM risk. In this review, HR > 1.0 was reported more frequently in women. European studies such as EPIC-InterAct (35) and Ericson et al. (36) found modest associations in women, while studies reported no or inverse associations, especially in men (37, 38).

### 4.4 Limitations

The interpretation of these results must take into account important limitations. First, the evidence derives mainly from observational cohort studies, which precludes causal inference and may be affected by residual confounding from lifestyle, socioeconomic, or dietary factors not fully captured in adjusted models. Second, the number of studies reporting sex-specific estimates remains limited, reducing statistical power and restricting generalisability, especially beyond Western and Asian populations where most cohorts were conducted. Third, exposures were defined heterogeneously (e.g., intake quintiles, daily servings, cooking frequency), complicating direct comparisons and partly explaining inconsistencies between cohorts. For this reason, no harmonization or dose re-expression was attempted, as such procedures would require unverifiable assumptions and loss of methodological detail. Another major limitation is the absence of formal sex-interaction tests in most primary studies. Reported differences were generally inferred from hazard ratios, an indirect approach that may either over- or underestimate effect modification. These signals should therefore be regarded as exploratory and hypothesis-generating rather than confirmatory. Finally, although estimates were extracted directly from the original studies, differences in exposure contrasts and covariate adjustments could lead to misinterpretation. To mitigate this, uniform annotation of contrast units, adjustment models, and significance thresholds was added to all tables and figures. In this context, the present review should be viewed as complementary to the federated meta-analysis by Li et al. (17), which found no overall sex interaction. Whereas pooled analyses provide robust standardized estimates, this narrative synthesis preserves study-specific contrasts and highlights potential sex-specific patterns and biological plausibility, pointing to areas where future pooled analyses with pre-specified interaction testing are needed.

## 5 Conclusions

The consumption of RM and PM is associated with an increased risk of T2DM, with more pronounced and consistent effects observed in men, particularly with regard to PM. WM shows weaker and less consistent associations, often with higher risk estimates in women. Taken together, our conclusions do not contradict but rather complement the federated meta-analysis by Li et al. (17). Their harmonized, pooled estimates indicate no overall sex interaction, whereas the present cohort-level compilation underscores heterogeneity in sex-specific risk patterns and plausible mechanisms. These observations warrant targeted, pre-specified interaction analyses in future pooled studies to determine whether and under what conditions sex meaningfully modifies meat-T2DM associations. Current findings suggest that sex-specific differences may be relevant when considering dietary strategies for T2DM prevention, but definitive changes to guidelines should await stronger confirmatory evidence (Table 3).

**Table 3:** Take-home messages and practical implications.

**Key Point**	**Details/Implications**
Red and processed meat	Consistently linked to higher T2DM risk, especially in men; current guidelines to limit intake remain appropriate for both sexes.
Gender differences	Biological and behavioral factors (hormones, fat distribution, iron metabolism, eating habits) may explain sex-specific susceptibilities; evidence remains exploratory.
White meat	Associations weaker and inconsistent; modest increases more often in women; no clear harmful or protective effect.
Cooking methods	High-temperature cooking (grilling, frying) increases exposure to HCAs and AGEs, which may impair insulin sensitivity; evidence limited.
Exposure heterogeneity	Use of quintiles, servings/day, or cooking frequency complicates comparability; results should be interpreted qualitatively.
Sex–interaction testing	Rarely reported; observed differences are hypothesis-generating, not confirmatory.
Regional patterns	Risk weaker in Asian cohorts (lower RM intake, more fish/soy) and stronger in Western cohorts (higher RM/PM intake).
Practical implications	No immediate need for sex-specific guidelines; refinement may be considered once stronger evidence from pooled analyses with pre-specified interaction testing is available.

RM, red meat; PM, processed meat; WM, white meat; T2DM, type 2 diabetes mellitus; HCAs, heterocyclic amines; AGEs, advanced glycation end-products.
