# Supplementary material for: Gender-specific effects of meat consumption on type 2 diabetes: a narrative review of prospective cohort studies
Source: Front Nutr. 2025 Nov 21;12:1665566. doi: 10.3389/fnut.2025.1665566 (PMC12679882; doi:10.3389/fnut.2025.1665566)
Supplement: Supplementary file 1 [file Table_1.DOCX]

**Supplementary Table S1. Summary of Exposure Definitions, Covariates, and Effect Measures Across Included Studies**

| Study (First Author, Year) | Risk Contrast for Main Estimate | Main Covariates in Fully Adjusted Model | Effect Measure (OR/HR) |
| --- | --- | --- | --- |
| Vang, 2008 | ≥1 serving/week vs never (vegetarian) | Age, sex, BMI, energy intake, physical activity, education | OR |
| Steinbrecher, 2010 | Q5 vs Q1 (quintiles of intake) | Age, survey year, energy intake, BMI, physical activity, smoking, education, alcohol, fibre, hypertension, family history diabetes | HR |
| EPIC-InterAct, 2013 | Per 50 g/day increment (continuous) | Age, centre, energy intake, BMI, education, physical activity, smoking, alcohol, fruit & veg, fibre, processed/red/total meat | HR |
| Ericson, 2013 | Q5 vs Q1 (quintiles of intake) | Age, energy intake, BMI, alcohol, smoking, education, physical activity, fat, carbohydrates, fibre, processed meat | HR |
| Kurotani, 2013 | Q4 vs Q1 (quartiles of intake) | Age, area, total energy, BMI, physical activity, smoking, alcohol, family history diabetes, hypertension, coffee, folate, magnesium | OR |
| Pan, 2013 | Q5 vs Q1 (quintiles of intake) | Age, energy intake, BMI, physical activity, alcohol, smoking, family history diabetes, race, menopausal status (women), other diet | HR |
| Liu, 2018 | >15 vs <4 times/month of open-flame/high-temp cooking | Age, energy intake, BMI, physical activity, alcohol, smoking, family history diabetes, race, menopausal status (women), total intake of red meat, chicken, fish, AHEI | HR |
| Gu, 2023 | Q5 vs Q1 (quintiles of intake) | Age, energy intake, physical activity, alcohol, smoking, race, menopausal status, total energy, other dietary factors (excluding BMI) | HR |

This supplementary table details, for each study included in the main analysis, the specific risk contrast (e.g., “Q5 vs Q1”, “per serving/day increment”, “≥1/week vs never”) and the main covariates included in the most fully adjusted model, and the type of effect measure used (OR or HR).
